# Supplementary material for: Global, asynchronous partial sweeps at multiple insecticide resistance genes in Aedes mosquitoes
Source: Nat Commun. 2024 Jul 24;15:6251. doi: 10.1038/s41467-024-49792-y (PMC11269687; doi:10.1038/s41467-024-49792-y)
Supplement: Supplementary file 3 — Description of Additional Supplementary Files [file 41467_2024_49792_MOESM3_ESM.pdf]

Description of Additional Supplementary Files

Supplementary Data 1. Aedes aegypti samples used in genomic analyses.

GST sweep indicates individuals with two copies of the GST haplotype, while * indicates individuals missing or heterozygous at one SNP. For other mutations wt = susceptible wild type.

Supplementary Data 2. Aedes albopictus samples used in genomic analyses.

wt = susceptible homozygous wild type genotype for F1534C polymorphism.

Supplementary Data 3. Number screened and frequencies of each VSSC allele in Aedes aegypti

Populations are the same as in Supplementary Data 1.

Supplementary Data 4. Number screened and frequencies of each VSSC allele in Aedes albopictus

Populations are the same as in Supplementary Data 2.

Supplementary Data 5. List of SNP positions on chromosome 3 strongly associated with region 'ii' in Fig 3(b,c)

Supplementary Data 6. List of genes indicated in Fig 5d (i-v).

Supplementary Data 7. List of SNP positions on chromosome 2 strongly associated with region 'i' in Fig 3(b,c)

Supplementary Data 8. List of genes indicated in Fig 6d (i-ii). (ii) Lists the 15 glutathione S-transferase (GST) genes

Supplementary Data 9. List of genes indicated in Fig 9

CHR = chromosome. POS = position on chromosome. LOC = gene identity. Symbol = symbol used in Fig 9.

Supplementary Data 10. List of genes indicated in Fig 10g

CHR = chromosome and contig. POS = position on contig. LOC = gene identity. Symbol = symbol used in Fig 10g.

Supplementary Code 1

Code used in processing, analysis, and plotting
