# Supplementary material for: Global, asynchronous partial sweeps at multiple insecticide resistance genes in Aedes mosquitoes
Source: Nat Commun. 2024 Jul 24;15:6251. doi: 10.1038/s41467-024-49792-y (PMC11269687; doi:10.1038/s41467-024-49792-y)
Supplement: Supplementary file 6 — Reporting Summary [file 41467_2024_49792_MOESM6_ESM.pdf]

nature portfolio

Corresponding author(s): Thomas L Schmidt

Last updated by author(s): Mar 26, 2024

Reporting Summary

Nature Portfolio wishes to improve the reproducibility of the work that we publish. This form provides structure for consistency and transparency in reporting. For further information on Nature Portfolio policies, see our Editorial Policies and the Editorial Policy Checklist.

Statistics

For all statistical analyses, confirm that the following items are present in the figure legend, table legend, main text, or Methods section.

n/a	Confirmed	
☐	☒	The exact sample size (*n*) for each experimental group/condition, given as a discrete number and unit of measurement
☒	☐	A statement on whether measurements were taken from distinct samples or whether the same sample was measured repeatedly
☐	☒	The statistical test(s) used AND whether they are one- or two-sided<br>*Only common tests should be described solely by name; describe more complex techniques in the Methods section.*
☒	☐	A description of all covariates tested
☐	☒	A description of any assumptions or corrections, such as tests of normality and adjustment for multiple comparisons
☐	☒	A full description of the statistical parameters including central tendency (e.g. means) or other basic estimates (e.g. regression coefficient) AND variation (e.g. standard deviation) or associated estimates of uncertainty (e.g. confidence intervals)
☐	☒	For null hypothesis testing, the test statistic (e.g. *F*, *t*, *r*) with confidence intervals, effect sizes, degrees of freedom and *P* value noted<br>*Give P values as exact values whenever suitable.*
☒	☐	For Bayesian analysis, information on the choice of priors and Markov chain Monte Carlo settings
☒	☐	For hierarchical and complex designs, identification of the appropriate level for tests and full reporting of outcomes
☒	☐	Estimates of effect sizes (e.g. Cohen's *d*, Pearson's *r*), indicating how they were calculated

Our web collection on statistics for biologists contains articles on many of the points above.

Software and code

Policy information about availability of computer code

Data collection	No software was used to collect data
Data analysis	All software used to analyse data are listed and cited in the Methods section

For manuscripts utilizing custom algorithms or software that are central to the research but not yet described in published literature, software must be made available to editors and reviewers. We strongly encourage code deposition in a community repository (e.g. GitHub). See the Nature Portfolio guidelines for submitting code & software for further information.

Data

Policy information about availability of data

All manuscripts must include a data availability statement. This statement should provide the following information, where applicable:

- Accession codes, unique identifiers, or web links for publicly available datasets
- A description of any restrictions on data availability
- For clinical datasets or third party data, please ensure that the statement adheres to our policy

Raw .fq files and relevant metadata for 934 mosquitoes will be available from the NCBI SRA following publication. Code used in processing, analysis, and plotting will be available from a Dryad repository. Source data will be provided with this paper, accessible via the Dryad repository.

Research involving human participants, their data, or biological material

Policy information about studies with [human participants or human data](). See also policy information about [sex, gender (identity/presentation), and sexual orientation]() and [race, ethnicity and racism]().

Reporting on sex and gender	N/A
Reporting on race, ethnicity, or other socially relevant groupings	N/A
Population characteristics	N/A
Recruitment	N/A
Ethics oversight	N/A

Note that full information on the approval of the study protocol must also be provided in the manuscript.

Field-specific reporting

Please select the one below that is the best fit for your research. If you are not sure, read the appropriate sections before making your selection.

☐ Life sciences ☐ Behavioural & social sciences ☒ Ecological, evolutionary & environmental sciences

For a reference copy of the document with all sections, see [nature.com/documents/nr-reporting-summary-flat.pdf]()

Ecological, evolutionary & environmental sciences study design

All studies must disclose on these points even when the disclosure is negative.

Study description	Genomic analysis of Aedes mosquitoes
Research sample	934 mosquitoes from across the world. These include Aedes aegypti and Aedes albopictus. These were collected from the field, except for the New Mexico sample which was lab-reared. In some cases, eggs were collected, and these were then hatched in the laboratory before sequencing the larva or adult.
Sampling strategy	Samples were collected periodically over >10 years. We aimed for >15 samples per population but this was not always attainable. These sample sizes are standard for the genomics literature in which n>3 or n>4 may be sufficient. Our study had a large sample size than many comparable studies that analyse whole genomes, and by using a reduced representation approach we have been able to use a large sample size.
Data collection	Samples were processed and sequenced by the authors. Authors collected of the most samples, other samples were collected by those listed in the Acknowledgements. Sample data were stored in Excel spreadsheets, recording date and lat/lon location where possible.
Timing and spatial scale	The spatial scale was global. Mosquitoes were collected by a variety of means, either as adults or juveniles. Samples were collected from 2012 to 2020, dates are listed in Supplementary Data 1 and 2. For sampling each population, we aimed to select single individuals separated by 50-100 m, to limit the number of close kin sampled. Sampling was conducted at any times possible due to COVID-19 restrictions which limited mobility.
Data exclusions	We excluded samples that were sequenced but had insufficient reads.
Reproducibility	We ran latent factor mixed models on two different data sets, corresponding to two VSSC mutations, and these produced almost identical patterns. All our code is reproducible, rerunning the data analysis from scratch will produce identical results.
Randomization	We allocated samples into groups based on geographical location.
Blinding	Blinding was not relevant as our analyses required prior knowledge of which mosquitoes were from which populations.

Did the study involve field work? ☒ Yes ☐ No

Field work, collection and transport

Field conditions	Samples were collected from Papua New Guinea throughout the year, under varying field conditions. As the samples were specifically used for genomics rather than phenotyping, these conditions were not considered as important for the study.

Location	Sampling was conducted at several locations across Papua New Guinea, as well as additional sampling from Kenya, Yogyakarta, Timor-Leste, and Tonga.
Access & import/export	All sampling was conducted with relevant local approvals. As samples are invasive pests, these did not require special permits. All samples were transported dead following standard importation protocols into Australia. The exception were the samples from New Mexico, which were imported as live eggs under the Department of Agriculture and Water Resources Permit to import conditionally non-prohibited goods: Permit No. 0002631825 and Department of the Environment and Energy NON-CITES permit PWS2019-AU-001275.
Disturbance	No disturbances were caused by the study.

Reporting for specific materials, systems and methods

We require information from authors about some types of materials, experimental systems and methods used in many studies. Here, indicate whether each material, system or method listed is relevant to your study. If you are not sure if a list item applies to your research, read the appropriate section before selecting a response.

Materials & experimental systems

n/a	Involved in the study
☒	☐ Antibodies
☒	☐ Eukaryotic cell lines
☒	☐ Palaeontology and archaeology
☐	☒ Animals and other organisms
☒	☐ Clinical data
☒	☐ Dual use research of concern
☒	☐ Plants

Methods

n/a	Involved in the study
☒	☐ ChIP-seq
☒	☐ Flow cytometry
☒	☐ MRI-based neuroimaging

Animals and other research organisms

Policy information about studies involving animals; ARRIVE guidelines recommended for reporting animal research, and Sex and Gender in Research

Laboratory animals	No lab animals
Wild animals	Aedes aegypti and Aedes albopictus mosquitoes, collected from populations worldwide. Collections involved either BioGents Sentinel traps, or sweep-nets, or ovitraps to collect eggs, or larvae collected from containers directly. All samples were killed immediately after sampling if adults or larvae, or if eggs they were transported to the laboratory and reared in the lab before being killed as larvae or adults.
Reporting on sex	Sex data were not recorded. But these are not relevant for this study, as very mionor genomic differences are expected between the sexes.
Field-collected samples	This did not involve samples collected from the field and kept alive.
Ethics oversight	No ethical approvals or guidance was required.

Note that full information on the approval of the study protocol must also be provided in the manuscript.

Plants

Seed stocks	No plants were used.
Novel plant genotypes	No plants were used.
Authentication	No plants were used.
